# Nebenwirkungen nach Immuncheckpointinhibitortherapie: Wissenswertes für Intensivmediziner:innen

**DOI:** 10.1007/s00063-023-01057-0

**Published:** 2023-09-03

**Authors:** Nina Buchtele, Hanna Knaus, Peter Schellongowski

**Affiliations:** 1 Intensivstation 13i2, Universitätsklinik für Innere Medizin I, Medizinische Universität Wien, Wien, Österreich; 2 Intensive Care in Hematologic and Oncologic Patients (iCHOP), Wien, Österreich; 3 Abteilung für Knochenmarktransplantation – KMT, Universitätklinik für Innere Medizin I, Medizinische Universität Wien, Wien, Österreich; 4 Universitätsklinik für Innere Medizin I, Intensivstation 13i2, Medizinische Universität Wien, Währinger Gürtel 18–20, 1090 Wien, Österreich

**Keywords:** Checkpointinhibitoren, Immuntherapie, Immunmediierte Nebenwirkungen, Unerwünschte Nebenwirkung, Organversagen, Checkpoint inhibition, Immunotherapy, Immune-related adverse events, Adverse reaction, Organ failure

## Abstract

Immuntherapien, und insbesondere Immuncheckpointinhibitoren, haben die Behandlung maligner Erkrankungen revolutioniert. Ihrem Wirkmechanismus geschuldet, der Aktivierung körpereigener T‑Zellen, sind jedoch auch häufig Nebenwirkungen die Folge einer Therapie. Sogenannte immunmediierte Nebenwirkungen („immune-related adverse event“, irAE) manifestieren sich als autoimmunologische Phänomene, können in jedem Organsystem auftreten und bis hin zu schwerem Organversagen führen. Aufgrund der zeitlichen Latenz von bis zu Monaten nach Verabreichung eines Checkpointinhibitors bis zur Erstmanifestation einer irAE ist es essenziell, bei entsprechender stattgehabter Therapie zu jedem Zeitpunkt an eine therapiespezifische Nebenwirkung zu denken. Bei beginnendem Organversagen ist das Absetzen des Checkpointinhibitors sowie der rasche Beginn mit einer Hochdosiskortisontherapie essenziell, die bei fehlendem Ansprechen um weitere Immunsuppressiva oder antiinflammatorische Therapieoptionen erweitert werden soll. Generell ist das Ansprechen auf Kortikosteroide und erweiterte Therapieoptionen gut und in diesem Sinne das Organversagen auch oft reversibel. Eine intensivmedizinische Betreuung mit etwaiger Notwendigkeit organunterstützender Therapien sollte dennoch nur streng nach Patient:innenwunsch sowie in enger Rücksprache mit den betreuenden Hämatoonkolog:innen erfolgen. Mit dem großen therapeutischen Nutzen, der häufigen Verwendung und dem vorhandenen Potenzial, in womöglich zukünftig auch kurativen Therapielinien zum Einsatz zu kommen, werden auch Intensivmediziner:innen häufiger mit irAEs nach Checkpointinhibitoren konfrontiert sein. Dementsprechend ist das Verstehen, Erkennen und Therapieren von Nebenwirkungen nach Immuntherapien zunehmend essenziell.

## Abstract (en)

Immunotherapies, and in particular checkpoint inhibitors, have revolutionized the treatment of cancer. However, due to their mechanism of action, the activation of the body’s own T cells, side effects are frequently associated with these therapies. So-called immune-related adverse events (irAEs) manifest as autoimmunological phenomena, can occur in any organ system, and even lead to severe organ failure. Due to the time latency of up to months after administration of a checkpoint inhibitor until the first manifestation of an irAE, it is essential to consider a therapy-specific adverse event at any time during therapy. In case of incipient organ failure, discontinuation of the checkpoint inhibitor and rapid initiation of high-dose corticosteroid therapy is essential, which, in the absence of response, should be extended by further immunosuppressive or anti-inflammatory therapies. In general, the response to corticosteroids and extended therapy options is good, and in this sense organ failure is often reversible. Nevertheless, intensive medical care with the possible need for organ-supporting therapies should only be provided strictly according to the patient’s wishes and in close consultation with the hematologist/oncologist in charge. Because of the great therapeutic benefit of immunotherapies, their frequent use, and potential to be used in curative lines of therapy in the future, intensive care physicians will also be confronted more frequently with irAEs after checkpoint inhibition. Accordingly, understanding, recognizing, and treating side effects after immunotherapies is increasingly essential for intensivists.

## Einführung

Immuntherapien sind eine Erfolgsgeschichte der modernen Medizin. Das Konzept, das Immunsystem gegen entartete Zellen, die es geschafft haben, den körpereigenen Abwehrmechanismen zu entkommen, zu reaktivieren, ist das Grundprinzip der Therapie. Unter anderem eben auch aufgrund solcher neuen Therapieoptionen hat sich die Kurz- und Langzeitprognose von Patient:innen mit malignen Erkrankungen deutlich verbessert, was auch zu einem Umdenken von Intensivmediziner:innen geführt hat, die nunmehr Krebspatient:innen durchweg liberaler intensivmedizinische Behandlung anbieten [1]; Bis zu einem Viertel der Intensivpatient:innen tragen heutzutage eine maligne Grunderkrankung mit sich [1, 2].

Neue Therapien bedeuten allerdings auch neue Nebenwirkungen, die bei Immuntherapien als potenziell „überschießende“ Immunreaktion auch zu mitunter lebensbedrohlichem Organversagen führen. Im Sinne der Reaktivierung des Immunsystems treten diese typischerweise als autoimmunologische Phänomene auf und können somit jedes Organsystem betreffen. Diese Übersichtsarbeit soll Intensivmediziner:innen dabei helfen, Immuntherapien zu verstehen, damit assoziierte Nebenwirkungen zu erkennen und diese behandeln zu können.

## Immuntherapien verstehen

Die Idee der Immuntherapie, außer Kraft gesetzte körpereigene Immunzellen zur Abwehr von entarteten Zellen zu reaktivieren, stammt aus dem 19. Jahrhundert; William Coley behandelte Krebspatient:innen erfolgreich mit Extrakten von inaktiviertem *Streptococcus pyogenes* und *Serratia marcescens* – nachdem er beobachtet hatte, dass Sarkompatient:innen ein besseres Outcome haben, wenn diese Erysipele hatten [3, 4]. Im 20. Jahrhundert konnte dann schließlich mithilfe neuer Technologien, wie die Verwendung von Knock-out-Mäusen, endgültig die notwendige Grundlage für das Verständnis der engen Verschränkung des körpereigenen Immunsystems mit dem Prozess der Entartung von Zellen geliefert werden [5].

Das Konzept von Immuntherapien rückte nun immer mehr T‑Zellen in den Mittelpunkt. Die T‑Zelle erkennt mittels ihres T‑Zell-Rezeptors Peptidantigene, die ihr im Rahmen der Antigenpräsentation über Major Histocompatibility Complex (MHC)-Moleküle präsentiert werden, und leitet damit eine adaptive Immunantwort ein. Die grobe Einteilung der T‑Zell-Subpopulation richtet sich nach ihrem exprimierten Korezeptor: CD4 oder CD8. Diese Korezeptoren sind im Rahmen der Immunantwort essenziell, um die Bindung an MHC-I-Moleküle (CD8-T-Zellen), oder MHC-II-Moleküle (CD4-T-Zellen) an den T‑Zell-Rezeptor zu ermöglichen. Weitere Korezeptoren können die Aktivierung der T‑Zelle verstärken (z. B. CD28) oder auch inhibieren (z. B. cytotoxic T lymphocyte antigen 4 (CTLA4) und programmed cell death 1 (PD1); [6]). Letztere stellen auch die wesentlichen Angriffspunkte der Checkpointinhibitoren zur Krebstherapie dar.

Ein anderes Konzept der Immuntherapie verfolgt der adoptive T‑Zell-Transfer, das über den Transfer von (in den meisten Fällen körpereigenen, also autologen) Zellen definiert ist, die zunächst dem Körper entnommen, ex vivo modifiziert und anschließend reinfundiert werden. Das historisch ältere Therapiekonzept – jedoch noch nicht in der klinischen Praxis verwendete – ist die Applikation von tumorinfiltrierenden Lymphozyten (TIL), die aus einer Gewebsprobe des Tumors des Patienten entnommen, isoliert, expandiert und autolog reinfundiert werden. Das historisch jüngere Therapiekonzept – das mittlerweile aus der modernen Krebstherapie nicht mehr wegzudenken ist – basiert auf der genetischen Modifizierung von entnommenen T‑Zellen mittels synthetischen antigenspezifischen Rezeptoren, den „chimeric antigen receptors“: die CAR-T-Zell Therapie [7].

Neben Checkpointinhibitoren und dem adoptiven T‑Zell-Transfer zählen auch Tumorvakzinen zu den Immuntherapien. Hierbei werden prophylaktische von therapeutischen Vakzinen unterschieden. Erstere verhindern die Primärinfektion mit einem onkogenen Virus (z. B. Hepatitis B oder humanes Papillomavirus) – als Impfstoff wird eine modifizierte, inaktivierte Form des entsprechenden Virus appliziert. Letztere kommen bei bereits bestehender Krebserkrankung zum Einsatz – als Impfstoff werden modifizierte tumorassoziierte Antigene verwendet, wobei meist autologe Tumorzellen entnommen, mit einem Adjuvans oder Virus versetzt und anschließend appliziert werden [8].

Nebenwirkungen nach Immuntherapien sind größtenteils auf den gewünschten Effekt zurückzuführen: eine Stimulierung und Aktivierung des Immunsystems zur erfolgreichen Tumorbekämpfung. Die numerisch am häufigsten verwendete Methode zur Immuntherapie sind Checkpointinhibitoren, weswegen fortführend in dieser Übersichtsarbeit auf diese Substanzklasse eingegangen wird.

## Entwicklungen im Bereich der Immuntherapie

**Figure 1:**
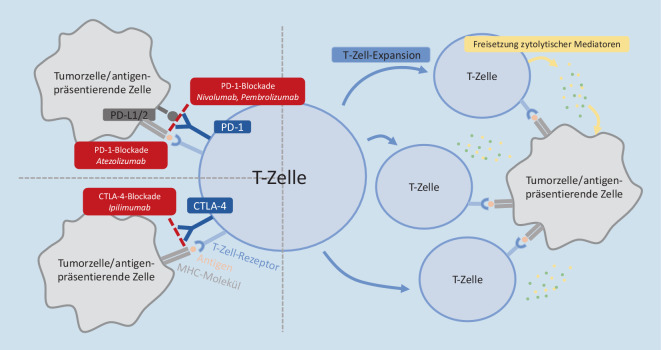

Im Rahmen der T‑Zell-Aktivierung verhindern sog. Checkpointrezeptoren eine Hyperaktivierung der Immunantwort, die somit als Regulator der Zellaktivierung zu sehen sind. Die potentesten und damit auch im Kontext der Immuntherapie hierfür relevantesten Vertreter sind die Rezeptoren CTLA4 und PD1; Abb. 1). Mit dem zunehmenden besseren Verständnis der engen Interaktion von Tumorzellen mit dem Immunsystem wurde somit die Idee der Checkpointinhibitoren geboren: 1996 erschien die erste Studie mit vielversprechenden Ergebnissen zum Einsatz eines CTLA4-Inhibitors zur immunmediierten Therapie von Tumoren im Mausmodell [9]. In darauffolgenden Studien wurde allerdings auch festgestellt, dass eine große Variabilität in den Ansprechraten unterschiedlicher Tumorentitäten besteht. Dennoch hinderte das nicht die Bestrebungen die Idee der Immuntherapie weiterzuentwickeln und letzten Endes klinische Studien durchzuführen – mit Erfolg [10]. Ipilimumab, ein humaner monoklonaler IgG1_κ_-anti-CTLA4-Antikörper, erlangte 2011 als erster Checkpointinhibitor seine Zulassung zur Behandlung des nichtresektablen Melanoms im Stadium III/IV. Die zugrunde liegenden klinischen Daten zeigten, dass die Therapie mit Ipilimumab zu einer Lebenszeitverlängerung von 3,6 Monaten führte – bei einem medianen Überleben von 10 Monaten in der Interventionsgruppe [11]. Weiterführende Langzeitstudien zeigten anschließend, dass bei ipilimumabbehandelten Melanompatient:innen nach etwa 3 Jahren ein Plateau in der Überlebenskurve erreicht wird – bei einer Überlebensrate von 20–25 %. Dementsprechend profitiert jeder 4. bis 5. Patient auch langfristig erheblich von einer Therapie mit Ipilimumab [12].

Die Euphorie der erfolgreichen Anti-CTLA4-Immuntherapie wurde allerdings relativ schnell gebremst – Ergebnisse bei Patient:innen mit Nierenzellkarzinom, nichtkleinzelligem und kleinzelligem Lungenkarzinom sowie Prostatakarzinom waren eher ernüchternd [13–16]. Ergebnisse weiterer CTLA4-Inhibitoren, wie Tremelimumab, konnten ebenso die Erwartungen nicht erfüllen. Dementsprechend verbleibt Ipilimumab für die Therapie des nichtresektablen Melanoms im Stadium III/IV als einziger Vertreter der CTLA4-Inhibitoren, die heute zur klinischen Anwendung kommen.

Nahezu zeitgleich zur Entwicklung von CTLA4-Inhibitoren begannen Bestrebungen, den zweiten prominenten Vertreter der wichtigen Checkpointrezeptoren – PD1 und dessen homologe Liganden PDL1 und PDL2 an antigenpräsentierenden oder Tumorzellen voranzutreiben. Zusammenfassend spricht man hier von der *PD1-Achse*. Nach vielversprechenden präklinischen Studien wurden 2010 erstmals humane Daten zur Verabreichung von MDX-1106 (später Nivolumab benannt), einem humanen monoklonalen gegen PD1 gerichteten IgG4-Antikörper, in 39 Patienten mit unterschiedlichen Tumorentitäten in fortgeschrittenem Stadium präsentiert [17]. Vier Jahre später folgte die Erstzulassung von Nivolumab sowie von Pembrolizumab, einem weiteren PD1-Blocker, zur Behandlung des fortgeschrittenen Melanoms. Kurze Zeit später konnte auch ein Überlebensvorteil von Pembrolizumab im Vergleich zum CTLA4-Inhibitor Ipilimumab in dieser Tumorentität gezeigt werden [18]. In den folgenden Jahren folgten zahlreiche Zulassungen der beiden PD1-Blocker Nivolumab und Pembrolizumab für weitere Tumorentitäten. Im Jahr 2016 wurde schließlich der erste PDL1-Hemmer (Atezolizumab) zugelassen [19], gefolgt von Zulassungen von Avelumab und Durvalumab in 2017. Zusammenfassend gab es entsprechend eine Flut an Zulassungen von Checkpointinhibitoren der PD1-Achse für fortgeschrittene Tumorerkrankungen – die nicht ganz unkritisch beäugt wurde und wird.

## Zulassung neuer Immuntherapien

Von 2015–2022 erlangten 7 PD-1- bzw. PD‑L1-Checkpointinhibitoren eine Zulassung durch die FDA in mehr als 85 Indikationen für onkologische Erkrankungen – eine immens hohe Anzahl verglichen mit anderen Wirkstoffklassen. Seitens der Pharmaindustrie herrscht ein großes Interesse an raschen Zulassungen, was sich letzten Endes in der Durchführung nichtkoordinierter klinischer Studien widerspiegelte. In beinahe der Hälfte der Indikationen für Checkpointinhibitoren wurde die jeweilige Zulassung über den „accelerated approval pathway“ (‚Programm zur beschleunigten Zulassung‘) erlangt [20]. Dieser wurde 1992 als Antwort auf die HIV/AIDS-Krise ins Leben gerufen, um raschere Zulassungen für Medikamente zu ermöglichen, die zur Behandlung *schwerwiegender Erkrankungen* dienen und einen *ungedeckten medizinischen Bedarf* erfüllen [21]. Der große Unterschied zur ‚traditionellen‘ Zulassung ist, dass als Endpunkt der Zulassungsstudie anstatt einer direkten Messung des klinischen Nutzens (wie z. B. Mortalität) lediglich ein Surrogatmarker herangezogen wird (wie z. B. Gesamtansprechrate [„overall response rate“, ORR] oder die Dauer des Ansprechens [„duration of response“, DOR]) – und das meistens in einem einarmigen Studiendesign. Nach erfolgter Zulassung über diesen „accelerated approval pathway“ sind die Arzneimittelhersteller verpflichtet, weiterführende Studien (Phase-IV-Studien) durchzuführen, um den erwarteten klinischen Nutzen zu bestätigen. Manche davon benötigen allerdings länger als eine Dekade, um abgeschlossen zu werden; eine Zeit, in der das Medikament in der klinischen Routine verabreicht werden kann und dementsprechend hohe Kosten verursacht, die dem Gesundheitssystem zur Last fallen, bevor die Zulassung im Fall eines fehlenden nachgewiesenen klinischen Nutzen entzogen wird [20].

Ein weiterer Kritikpunkt dieses Zulassungswegs ist, dass klinische Studien somit zunächst bei Patient:innen mit refraktären Erkrankungen ohne weitere Therapieoptionen durchgeführt werden – da hier ebendieser „unmet medical need“ besteht. Das führt in weiterer Folge jedoch dazu, dass weniger Studien bei Patient:innen in früheren Therapielinien durchgeführt werden – da hier ein aufwendigeres, 2‑armiges Studiendesign von Nöten wäre, obwohl ebendiese Patient:innen ebenso von der Therapie profitieren könnten. Das ist unter anderem auch eine Erklärung, weswegen bis dato beinahe alle Indikationen zur Immuntherapie im nichtkurativen Setting liegen. Entsprechend ist allerdings auch zu erwarten, dass in Zukunft Checkpointinhibitoren auch in früheren Therapielinien zum Einsatz kommen werden – eine Entwicklung, die vor allem für Intensivmediziner:innen von hoher Relevanz ist, wenn etwaige Entscheidungen bezüglich einer Therapielimitierung gefällt werden.

## Welche Patient:innen erhalten Checkpointinhibitoren und was bedeutet das für Intensivmediziner:innen?

Die häufigsten Zulassungsindikationen für Checkpointinhibitoren umfassen solide Tumoren in fortgeschrittenen Tumorstadien (Tab. 1). Die zurzeit am häufigsten verwendeten Substanzen sind die PD-1/PD-L1-Hemmer Pembrolizumab Keytruda®, Merck, Kenilworth, NJ, USA; KEYNOTE Studien), Nivolumab (Opdivo®, Bristol-Myers Squibb, New York City, NY, USA, CheckMate-Studien), Atezolizumab (Tecentriq®, Roche, Basel, Schweiz) sowie der am längsten zugelassene Checkpointinhibitor zur CTLA4-Blockade Ipilimumab (Yervoy®, Bristol-Myers Squibb, New York City, NY, USA; [22]).

**Table 1:** 

Entität	Substanz
Kopf-Hals-Plattenepithelkarzinome	Pembrolizumab
	Nivolumab
Melanom	Ipilimumab
	Pembrolizumab
	Nivolumab
	Ipilimumab plus Nivolumab
Merkel-Zell-Karzinom	Pembrolizumab
	Avelumab
Hepatozelluläres Karzinom	Pembrolizumab
	Nivolumab
Nierenzellkarzinom	Nivolumab plus Ipilimumab
	Nivolumab
Zervixkarzinom	Pembrolizumab
Kleinzelliges Lungenkarzinom	Atezolizumab
	Nivolumab
Nichtkleinzelliges Lungenkarzinom	Pembrolizumab
	Atezolizumab
	Nivolumab
Triple negatives Mammakarzinom	Atezolizumab
Magenkarzinom	Pembrolizumab
Hodgkin-Lymphom	Pembrolizumab
Urothelkarzinom	Pembrolizumab
	Atezolizumab
	Avelumab
	Durvalumab
	Nivolumab

### Bedeutung für Intensivmediziner:innen

Aufgrund der stetig zunehmenden Verwendung von Checkpointinhibitoren in verschiedenen Tumorentitäten – in mittlerweile auch früheren Stadien der Erkrankung – wird es einerseits wahrscheinlicher, Patient:innen nach einer solchen Therapie auf der Intensivstation aufzunehmen, allerdings auch immer schwieriger als intensivmedizinisches Team, das womöglich nicht auf hämatoonkologische Grunderkrankungen spezialisiert ist, die Indikation und insbesondere die individuelle Prognose des Patienten bzw. der Patientin zu überblicken. Insgesamt sollten daher von Anfang an die betreuenden Onkolog:innen bzw. Hämatolog:innen in das Management miteinbezogen werden – auch bei der Festlegung etwaiger Therapieziele auf der Intensivstation. Letztere sollte insgesamt nach folgenden Punkten evaluiert werden:

1. der Wille und Wunsch des Patienten bzw. der Patientin bezüglich intensivmedizinischer Therapiemaßnahmen;
2. die Prognose einhergehend mit der zugrunde liegenden Erkrankung;
3. der Allgemein- und Leistungszustand des Patienten bzw. der Patientin vor und während des Krankenhausaufenthalts;
4. die Chance der Reversibilität des Organversagens, das eine intensivmedizinische Betreuung notwendig macht [23].

Der (auch vermeintliche) Patient:innenwunsch – insbesondere einer Ablehnung gegenüber intensivmedizinischer Maßnahmen – sollte selbstverständlich immer im Vordergrund jeglicher Entscheidung stehen und bestmöglich bereits vor Bestehen einer kritischen Krankheit diskutiert worden sein. In Bezug auf die individuelle Prognose des Patienten bzw. der Patientin ist zu berücksichtigen, dass Patient:innen, die Checkpointinhibitortherapien erhalten, zwar nur sehr selten in einem kurativen Setting behandelt werden, aber dennoch eine lange Lebenserwartung mit zufriedenstellender Lebensqualität haben können. Dementsprechend kann somit auch eine intensivstationäre Behandlung ohne Einschränkung der intensivmedizinischen Ressourcen („full-code ICU management“) sinnvoll sein. Bezüglich der Reversibilität des Organversagens bietet sich zur besseren Einschätzung auch ein *ICU Trial* an, also ein zeitlich limitiertes *Full-code-Management*, das eine Reevaluierung nach wenigen, z. B. 5, Tagen bezüglich der Dynamik des Organversagens vorsieht – was insbesondere bei checkpointinhibitorinduzierten Nebenwirkungen, die oft nach kurzer Therapiedauer bereits Besserungen zeigen, hilfreich sein kann.

Insgesamt sollte in Bezug auf Therapieziellimitierungen unbedingt berücksichtigt werden, dass diese einerseits ein dynamischer Prozess sind und regelmäßig – in beide Richtungen – reevaluiert werden müssen und auch eine vorab festgelegte Therapiebegrenzung keine Kontraindikation zur intensivstationären Aufnahme darstellt [23, 24].

## Keine Wirkung ohne Nebenwirkung

Durch die Beeinflussung des Immunsystems durch Checkpointinhibitoren manifestieren sich Nebenwirkungen durch diese Therapien typischerweise als immunmediierte Nebenwirkung („immune-related adverse event“, irAE) – im Sinne einer überschießenden Immunreaktion. Die individuellen Auswirkungen spiegeln Autoimmunphänomene wider, die jedes Organsystem betreffen können, allerdings wirkstoffbezogene Häufungsgipfel zeigen.

### Klassifizierung der immunmediierten Nebenwirkungen in Schweregrade

Immunmediierte Nebenwirkungen werden organspezifisch gemäß der Toxizität in 4 Schweregrade eingeteilt. Je nach betroffenem Organ erfolgt die Einteilung anhand laborchemischer Parameter (z. B. Transaminasen bei hepatischer Manifestation), klinisch (z. B. Ausmaß der Veränderung bei dermatologischer/kutaner Manifestation) bzw. anhand der notwendigen supportiven Maßnahmen (z. B. O_2_-Insufflation bei pulmonaler Manifestation). Letzten Endes ist die Bestimmung des Schweregrads relevant, da sich auch daraus die entsprechende empfohlene Therapie ableitet.

Generell umfassen irAE von Grad I milde Nebenwirkungen, worunter unter engmaschiger Kontrolle die Immuntherapie fortgeführt werden kann und keine spezifische Therapie erforderlich ist. Immunmediierte Grad-II-Nebenwirkungen beinhalten moderate Toxizitäten, bei denen eine Pausierung der Immuntherapie bis zum Rückgang auf (zumindest) eine Grad-I-Toxizität erwogen werden soll. Bei Auftreten von Grad-III-irAE, im Sinne von schweren Nebenwirkungen sollte neben der Beendigung der Immuntherapie eine immunsuppressive Therapie mit Kortikosteroiden begonnen werden und bei Grad-IV-irAE handelt es sich um lebensbedrohliche Nebenwirkungen, bei denen neben der Hochdosiskortikosteroidtherapie eine permanente Beendigung der Immuntherapie empfohlen wird. Insgesamt sollten Intensivmediziner:innen grob orientierend mit der Klassifikation der Grad-III- und Grad-IV-irAE vertraut sein, die für die entsprechenden Organsysteme in Tab. 2 dargestellt ist [25].

**Table 2:** 

Organsystem	Definition Grad III	Definition Grad IV
Haut – Dermatitis/bullöse Dermatose	Hautmanifestationen > 30 % „body surface area“ mit Einschränkung von täglichen Lebensaktivitäten	Wie Grad III einhergehend mit schweren/lebensbedrohlichen Flüssigkeits- und Elektrolytstörungen
Gastrointestinaltrakt – Kolitis	Zunahme um ≥ 7 Stuhlgängen pro Tag gegenüber dem Ausgangswert mit Einschränkung von täglichen Lebensaktivitäten	Wie Grad III einhergehend mit schweren/lebensbedrohlichen Flüssigkeits- und Elektrolytstörungen
Leber – Hepatitis	Transaminasen (AST, ALT) > 5 bis 20-fach und/oder Bilirubin 3 bis 10-fach der oberen Normgrenze *ode*r symptomatische Leberdysfunktion, Fibrose oder Reaktivierung einer chronischen Hepatitis	Transaminasen (AST, ALT) > 20-fach und/oder Bilirubin > 10-fach der oberen Normgrenze *oder* klinische Zeichen eines akuten Leberversagens (Aszites, Koagulopathie, Enzephalopathie, Koma)
Lunge – Pneumonitis	Radiographische Beteiligung von allen Quadranten oder > 50 % des Lungenparenchyms mit Notwendigkeit von O_2_-Insufflation und Einschränkung von täglichen Lebensaktivitäten	Lebensbedrohliche respiratorische Insuffizienz sowie Notwendigkeit der invasiven mechanischen Beatmung
Schilddrüse – Thyreoiditis	TSH > 10 mIU/l und Einschränkung von täglichen Lebensaktivitäten	
Nebennierenrinde – Nebenniereninsuffizienz	Morgendliches ACTH > 2-fach der oberen Normgrenze und Kortisol < 3 µg/dl und Einschränkung von täglichen Lebensaktivitäten	
Hypophyse – Hypophysitis	Veränderungen in ACTH, Kortisol bzw. TSH, fT4 passend zur primären Insuffizienz der kortikotropen oder thyreotropen Achse mit Einschränkung von täglichen Lebensaktivitäten	
Diabetes	Neu aufgetreten oder Verschlechterung eines bereits bestehenden mit Nüchternblutzucker 250–500 mg/dl	Neu aufgetreten oder Verschlechterung eines bereits bestehenden mit Nüchternblutzucker > 500 mg/dl
Gelenke – Arthritis	Starke Schmerzen mit Gelenksschwellung und Einschränkung von täglichen Lebensaktivitäten	
Muskel – Myositis	Schwere Muskelschwäche mit Einschränkung von täglichen Lebensaktivitäten	
Muskel – Polymyalgie	Schwere Steifigkeit und Schmerzen mit Einschränkung von täglichen Lebensaktivitäten	
Niere – Nephritis	Kreatinin > 3-fach des Ausgangswerts oder > 4 mg/dl	Dialysepflichtigkeit oder Kreatinin > 6-fach des Ausgangswert
Neurologisch – Myasthenia gravis	Positive Acetylcholinrezeptorantikörper, Antikörper gegen muskelspezifische Kinase (MuSK) oder Lipoprotein-related-4(LPR4)-Antikörper im Blut mit Einschränkung von täglichen Lebensaktivitäten *oder* Schluckstörungen, Schwäche der Gesichts- oder Atemmuskulatur, rasch progrediente Symptome *oder* moderat bis schwere generalisierte Schwäche	
Neurologisch – Guillain-Barré-Syndrom	Zytoalbuminäre Dissoziation im Liquor und deutliche Verlangsamung der peripheren Nervenleitgeschwindigkeit ± Antigangliosidantikörper mit einhergehender körperlicher Schwäche bzw. jeglichem Vorliegen von Schluckstörung, Schwäche in der Gesichtsmuskulatur oder Atemmuskulatur oder rasch Fortschreitende Schwäche	
Blutbildende Organe – hämolytische Anämie	Evidenz von Hämolyse (erhöhte LDH, erniedrigtes Haptoglobin, erhöhtes Bilirubin, erhöhte Retikulozyten) und Hämoglobin < 8,0 g/dl oder Notwendigkeit von Transfusionen	Wie Grad III einhergehend mit schweren/lebensbedrohlichen Komplikationen
Herz – Myokarditis, Perikarditis	Erhöhte Herzenzyme (Troponin, CK) ohne Hinweis auf Myokardischämie mit moderaten Symptomen oder neu aufgetretener Leitungsblockade im EKG	Wie Grad III mit schwerer Dekompensation und Notwendigkeit von i.v.-Medikation oder Intervention

*ACTH* Adrenocorticotropes Hormon, *ALT* Alanin-Aminotransferase, *AST* Aspartat-Aminotransferase, *CK* Creatin-kinase, *fT4* freies Thyroxin, *LDH* Laktatdehydrogenase, *TSH* Thyreoidea-stimulierendes Hormon

### Häufigkeit von Nebenwirkungen

**Figure 2:**
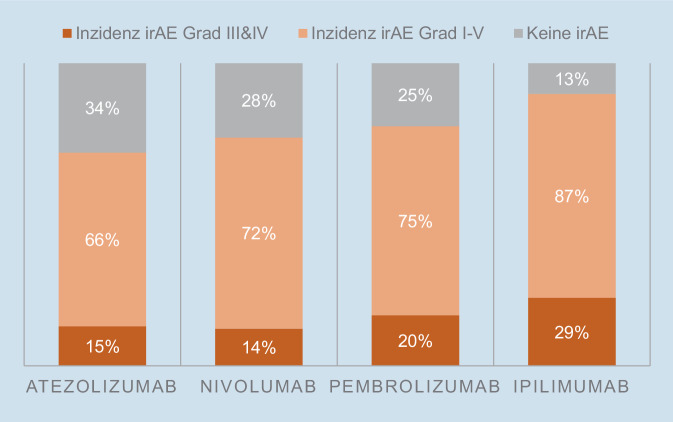

Die bis dato umfassendste Netzwerkmetaanalyse aus dem Jahr 2018, publiziert im *British Medical Journal*, analysierte Head-to-Head Phase-II- und -III-Studien mit Checkpointinhibitoren, um dahingehend deren Sicherheitsprofile zu vergleichen [26]. Folgende aufschlussreiche Daten über die am häufigsten verwendeten Checkpointinhibitoren wurde in dieser Arbeit präsentiert:

1. CTLA-4-blockierende Substanzen (Ipilimumab) rufen häufiger und schwerwiegendere irAE hervor als PD-1/PD-L1-blockierende Substanzen (Nivolumab, Pembrolizumab, Atezolizumab; Abb. 2).
2. Eine Kombinationstherapie mit verschiedenen Checkpointinhibitoren erhöht die Inzidenz und den Schweregrad von irAE im Vergleich zur Monotherapie.
3. PD-1/PD-L1-blockierende Substanzen haben ein besseres Sicherheitsprofil als konventionelle Chemotherapie.
4. Folgende Nebenwirkungen kamen nach Wirkstoff gehäuft vor:
  Atezolizumab: Hypothyreose, Erbrechen, Übelkeit;
  Pembrolizumab: Arthritis, Pneumonitis, Hepatitis;
  Nivolumab: Endokrine Toxizitäten:
  Ipilimumab: Haut, gastrointestinale Toxizitäten, Nephritis;
5. Für Pembrolizumab besteht ein dosisabhängiger Effekt für das Auftreten für irAE, nicht jedoch für andere Checkpointinhibitoren.

Diebis dato größte (retrospektive) Studie aus dem intensivmedizinischen Setting umfasste 112 Patient:innen aus 4 französischen Zentren, die innerhalb der letzten 60 Tage einen Checkpointinhibitor erhalten haben. Bei 29 Patient:innen (26 %) erfolgte die intensivstationäre Aufnahme aufgrund einer irAE, am häufigsten wurde eine Pneumonitis diagnostiziert (in 28 % der Fälle).

In diesem Kontext ist noch wichtig zu bedenken, dass Nebenwirkungen nach Immuntherapie im Median 40 Tage nach der Therapie auftreten, jedoch auch noch Jahre nach der Therapie auftreten können [27]. Dementsprechend sind vor allem spät auftretende Nebenwirkungen in kontrollierten Studien oft nicht abgebildet, da sie erst nach dem vordefinierten Follow-up manifest werden. Zudem muss bedacht werden, dass Studiensettings, wie sie dieser großen Netzwerkmetaanalyse zugrunde liegen, oft ein Patient:innenkollektiv mit weniger Komorbiditäten abbilden und vergleichbare Daten in dieser Größe und Qualität aus dem *Real-world*-Setting noch nicht ausreichend vorhanden sind [26].

### Erkennen und Diagnostizieren

**Figure 3:**
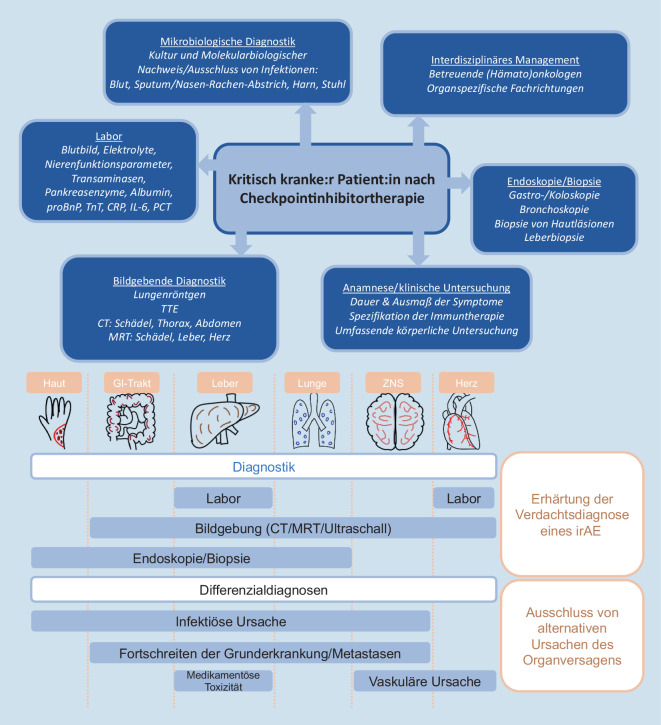

Aufgrund der Häufigkeit der mitunter schweren irAE nach Checkpointinhibitortherapie sollte bei jedem/jeder Patient:in nach Erhalt einer entsprechenden Therapie eine darauf beruhende Nebenwirkung differenzialdiagnostisch miteinbezogen werden. Auszuschließen ist hier bei allen Organsystemen I) ein Fortschreiten der Grunderkrankung und II) eine infektiöse Genese. Das diagnostische Work-up sollte entsprechende laborchemische Untersuchungen, mikrobiologische Diagnostik, Bildgebung und in manchen Fällen eine Biopsie umfassen. Empfehlenswert ist zusätzlich die kontinuierliche Miteinbeziehung der organfachspezifischen Experten, um klinische Befunde und Differenzialdiagnosen richtig zu interpretieren [25]. Abb. 3 gibt eine Übersicht über mögliche indizierte Abklärungen und Differenzialdiagnosen je nach Organsystem.

## Therapiestrategien

Die Therapie einer schweren irAE (Grad III und IV) basiert unabhängig vom betroffenen Organsystem auf einer Unterdrückung der Immunantwort. Als primäre Behandlungsstrategie wird eine Kortikosteroidtherapie empfohlen (Tab. 3; [25]). Sollte kein Ansprechen auf Kortikosteroide erkennbar sein, wird je nach betroffenem Organsystem eine Erweiterung der Therapie um weitere Immunsuppressiva (z. B. Mycophenolat-Mofetil, Azathioprin) oder primär antiinflammatorisch wirksame Substanzen empfohlen (z. B. Infliximab; Tab. 4). Je nach Organsystem werden unterschiedliche Therapiedauern einer initialen Kortisontherapie für die Definition der steroidrefraktären irAE verwendet. Eine Übersicht zur Dosierung der entsprechenden erweiternden Therapieoptionen ist z. B. der Tab. A2 aus Schneider et al., *J Clin Oncol 39:4073–4126* zu entnehmen. Sämtliche Therapieoptionen, sowohl die Erstlinientherapie mit Steroiden als auch die Zweitlinientherapien im steroidrefraktären Setting, basieren lediglich auf Expert:innenmeinungen und wurden bis dato nicht in randomisierten klinischen Studien evaluiert [25].

**Table 3:** 

Organsystem	Diagnose	Grad III	Tapering	Grad IV	Tapering
Haut	Dermatitis	1 mg/kgKG Prednisolon p.o.	> 4 Wochen	1–2 mg/kgKG Methyprednisolon i.v.	Nach Abklingen der Toxizität langsames Tapering
	Bullöse Dermatose	1–2 mg/kgKG Methyprednisolon i.v.	> 4 Wochen	1–2 mg/kgKG Methyprednisolon i.v.	>4 Wochen
Gastrointestinaltrakt	Kolitis	1–2 mg/kgKG Prednisolon p.o.	4–6 Wochen	1–2 mg/kgKG Methyprednisolon i.v.	4–6 Wochen
	Hepatitis	1–2 mg/kgKG Methyprednisolon i.v.	4–6 Wochen	2 mg/kgKG Methyprednisolon i.v.	4–6 Wochen
Lunge	Pneumonitis	1–2 mg/kgKG Methyprednisolon i.v.	4–6 Wochen	1–2 mg/kgKG Methyprednisolon i.v.	–
Endokrine Organe	Nebenniereninsuffizienz	Stressdosis Steroide: 50–100 mg Hydrokortison alle 6–8 h	Auf orale Erhaltungsdosis über 5–7 Tage	Stressdosis Steroide: 50–100 mg Hydrokortison alle 6–8 h	Auf orale Erhaltungsdosis über 5–7 Tage tapern
	Hypophysitis	Stressdosis Steroide: 50–100 mg Hydrokortison alle 6–8 h	Auf orale Erhaltungsdosis über 5–7 Tage	50–100 mg Hydrokortison alle 6–8 h	Auf orale Erhaltungsdosis über 5–7 Tage tapern
		Pulsdosis Steroide: 1–2 mg/kgKG Prednisolon p.o	Auf orale Erhaltungsdosis über 1–2 Wochen	1–2 mg/kgKG Prednisolon p.o	Auf orale Erhaltungs-dosis über 1–2 Wochen tapern
Muskuloskelettal	Arthritis	0,5–1 mg/kgKG Prednisolon p.o	4–6 Wochen	0,5–1 mg/kgKG Prednisolon p.o	4–6 Wochen
	Myositis	1 mg/kgKG Prednisolon p.o oder 1–2 mg/kgKG Methyprednisolon i.v.	Langsames Tapering	1 mg/kgKG Prednisolon p.o oder 1–2 mg/kgKG Methyprednisolon i.v.	Langsames Tapering
	Polymyalgie	40 mg Prednisolon p.o.	Langsames Tapering	40 mg Prednisolon p.o.	Langsames Tapering
Zentrales Nervensystem	Myasthenia gravis	0,5 mg/kgKG Prednisolon	Nach 3–4 Wochen	0,5 mg/kgKG Prednisolon	Nach 3–4 Wochen
	Guillain-Barré-Syndrom und periphere Neuropathie	2–4 mg/kgKG Methyprednisolon i.v.	4–6 Wochen	2–4 mg/kgKG Methyprednisolon i.v.	4–6 Wochen
Herz	Myokarditis und Perikarditis	1–2 mg/kgKG Prednisolon p.o. oder i.v.	4–6 Wochen	1–2 mg/kgKG Prednisolon p.o. oder i.v. bis hin zu 1g Methylprednisolon i.v.	Über 4–6 Wochen

**Table 4:** 

Organsystem	Diagnose	Erweiternde immunsuppressive Therapie
Haut	Bullöse Dermatose	Ab Grad III IVIG oder Rituximab als kortisonsparende Option
Gastrointestinaltrakt	Kolitis	Steroidrefraktär (72 h): Infliximab (TNF-α-Blocker), Vedolizumab (Integrinantagonist), Tofacitinib (JAK-Inhibitor), Ustekinumab (IL-12-Blocker), Stuhltransplantation
	Hepatitis	Steroidrefraktär (72 h): Azathioprin, Mycophenolat-Mofetil
Lunge	Pneumonitis	Steroidrefraktär (48 h): Infliximab, Mycophenolat-Mofetil, IVIG, Cyclophosphamid
Muskuloskelettal	Arthritis	Steroidrefraktär (2 Wochen): Methotrexat, Leflunomid, Hydroxychloroquin, Sulfasalazin, TNF-α- oder IL-6-Antagonisten
	Myositis	Plasmapherese, IVIG; steroidrefraktär (2 Wochen): Rituximab, TNF-α- oder IL-6-Antagonisten, Methotrexat, Azathioprin, Mycophenolat-Mofetil
	Polymyalgie	Methotrexat, IL-6-Antagonisten
Zentrales Nervensystem	Myasthenia gravis	Ab Grad II: Pyridostigmin; Ab Grad III: IVIG oder Plasmapherese; steroidrefraktär (nach klinischem Ermessen): Rituximab
	Guillain-Barré-Syndrom, periphere Neuropathie	Ab Grad II: IVIG oder Plasmapherese
Herz	Myokarditis, Perikarditis	Steroidrefraktär (nach Initialdosis): Mycophenolat-Mofetil, Infliximab, Antithymozytenglobulin, Abatacept (kostimulatorische Molekülblockade), Alemtuzumab (CD52-Blockade)

In der bereits erwähnten französischen Studie wurden 62 % der Patient:innen mit Kortikosteroiden behandelt; eine Zweitlinientherapie war lediglich in 10 % der Patient:innen notwendig, was eine hohe Ansprechrate auf Steroide suggeriert [28]. Ferner konnte gezeigt werden, dass Patient:innen mit irAE eine niedrigere 1‑Jahres-Mortalität im Vergleich zu Patient:innen nach Checkpointinhibitortherapie, die *nicht* aufgrund einer irAE eine intensivstationäre Behandlung benötigten, aufwiesen. Eine Metaanalyse in beinahe 20.000 Patient:innen zeigte toxizitätsbedingte Todesraten von 0,36 % (Anti-PD1) bis hin zu 1,23 % (Kombinationstherapie Anti-PD1/PD-L1 und Anti-CTLA4), die entsprechend als niedrig einzustufen sind [29].

Neben der Unterdrückung der Immunantwort, also der Therapie des zugrunde liegenden Problems, sollte unbedingt eine mikrobiologische Diagnostik und gegebenenfalls bei nicht auszuschließender infektiöser Ursache eine empirische antimikrobielle Therapie bedacht werden. Ein Review der European Society of Clinical Microbiology and Infectious Diseases (ESCMID) kam zu dem Schluss, dass Checkpointinhibitoren *per se* nicht das Infektionsrisiko erhöhen [30]. Eine Metaanalyse aus dem Jahr 2021 konnte in diesem Zusammenhang jedoch zeigen, dass Patient:innen mit Checkpointinhibitormonotherapie ein niedrigeres Infektionsrisiko haben als Patient:innen, die eine Kombinationstherapie oder eine Chemotherapie mit einem Checkpointinhibitor erhalten haben [31].

Bei der Therapie von irAE sollte in weiterer Folge in Betracht gezogen werden, dass es im Verlauf unter Kortisontherapie zu einer höheren Suszeptibilität für opportunistische Erreger, Pilzinfektionen und Virusreaktivierungen kommt und sich dementsprechend das Spektrum der infektiösen Erreger ändert [25, 30].

Zusätzliche intensivmedizinische Maßnahmen (wie z. B. Sauerstoffinsufflation) unterscheiden sich nicht von denen, mit denen Patient:innen ohne irAE unterstützt werden.

## Wie geht es weiter nach der kritischen Krankheit?

Schwere Nebenwirkungen nach Checkpointinhibitortherapie sind mit einer hohen Rate an Reversibilität und damit relativ niedriger Mortalität auf der Intensivstation von 17–28 % gemäß rezenter Literatur vergesellschaftet [28, 32, 33]. Entsprechend stellt sich für eine Vielzahl der Patient:innen und deren Behandler:innen die Frage, wem, womit und wann eine erneute Therapie verabreicht werden kann und soll. Hier steht nunmehr das potenzielle Fortschreiten der Krebserkrankung dem erneuten Auftreten einer schweren Nebenwirkung gegenüber, was somit abgewogen werden muss. Die Datenlage zu dieser spezifischen Fragestellung ist hierfür noch spärlich und klare Empfehlungen sind ausständig, wodurch der Entscheid zum Wiederbeginn einer Immuntherapie individualisiert erfolgen muss – auch unter Miteinbeziehung des Patient:innenwunschs. Französische Pharmakovigilanzdaten konnten zuletzt zeigen, dass rund 60 % der Patient:innen, bei denen eine Checkpointinhibitortherapie aufgrund einer irAE im Grad ≥ 2 beendet wurde, bei einer Re-Challenge keine erneute irAE erlitten haben [34]. Insgesamt ist die wichtige Frage nach dem sicheren aber potenziell lebensnotwendigen Wiederbeginn der Therapie in zukünftigen prospektiven Studien zu beantworten.

## Fazit für die Praxis

- Immunmediierte Nebenwirkungen (irAE) nach Therapie mit Checkpointinhibitoren sind häufig und können mitunter zu Organversagen führen, weswegen Intensivmediziner:innen mit deren Erkennen und der Therapie vertraut sein sollten.
- Immunmediierte Nebenwirkungen treten häufig in den ersten Wochen nach der Therapie auf, können jedoch auch erst nach Monaten manifest werden, weswegen Intensivmedizinier:innen bei entsprechender Anamnese irAE bedenken müssen.
- Die Therapie basiert auf einer Unterdrückung der durch Checkpointinhibitoren hervorgerufenen Immunantwort und umfasst entsprechend primär eine Hochdosiskortikosteroidtherapie und bei ausbleibender Besserung eine Ergänzung um weitere Immunsuppressiva bzw. antiinflammatorisch wirksame Medikamente.
- Die Indikation für eine intensivstationäre Betreuung richtet sich nach der Schwere des Organversagens. Da schwere irAE häufig gut auf die Einleitung einer Therapie ansprechen, sollte sich der Entscheid zur Intensivtherapie mit dem Patienten/der Patientin und dem betreuenden Onkologen/Onkologin in erster Linie auf den funktionellen Status des Patienten/der Patientin vor dem Organversagen stützen.
